# Self-Assembly of a Novel Pentapeptide into Hydrogelated Dendritic Architecture: Synthesis, Properties, Molecular Docking and Prospective Applications

**DOI:** 10.3390/gels10020086

**Published:** 2024-01-23

**Authors:** Stefania-Claudia Jitaru, Andra-Cristina Enache, Corneliu Cojocaru, Gabi Drochioiu, Brindusa-Alina Petre, Vasile-Robert Gradinaru

**Affiliations:** 1 Faculty of Chemistry, “Alexandru Ioan Cuza” University, 11 Carol I Bd., 700506 Iasi, Romania; jitaru.stefania@yahoo.com (S.-C.J.); gabi.drochioiu@gmail.com (G.D.); brindusa.petre@uaic.ro (B.-A.P.); robert.gradinaru@uaic.ro (V.-R.G.); 2 “Petru Poni” Institute of Macromolecular Chemistry, 41-A Grigore Ghica Voda Alley, 700487 Iasi, Romania; cojocaru.corneliu@icmpp.ro; 3 TRANSCEND-Regional Institute of Oncology, 700483 Iasi, Romania

**Keywords:** peptide, self-assembling, supramolecular gels, β-sheet, molecular docking, polyplexes

## Abstract

Currently, ultrashort oligopeptides consisting of fewer than eight amino acids represent a cutting-edge frontier in materials science, particularly in the realm of hydrogel formation. By employing solid-phase synthesis with the Fmoc/tBu approach, a novel pentapeptide, FEYNF-NH_2_, was designed, inspired by a previously studied sequence chosen from hen egg-white lysozyme (FESNF-NH_2_). Qualitative peptide analysis was based on reverse-phase high performance liquid chromatography (RP-HPLC), while further purification was accomplished using solid-phase extraction (SPE). Exact molecular ion confirmation was achieved by matrix-assisted laser desorption–ionization mass spectrometry (MALDI-ToF MS) using two different matrices (HCCA and DHB). Additionally, the molecular ion of interest was subjected to tandem mass spectrometry (MS/MS) employing collision-induced dissociation (CID) to confirm the synthesized peptide structure. A combination of research techniques, including Fourier-transform infrared spectroscopy (FTIR), fluorescence analysis, transmission electron microscopy, polarized light microscopy, and Congo red staining assay, were carefully employed to glean valuable insights into the self-assembly phenomena and gelation process of the modified FEYNF-NH_2_ peptide. Furthermore, molecular docking simulations were conducted to deepen our understanding of the mechanisms underlying the pentapeptide’s supramolecular assembly formation and intermolecular interactions. Our study provides potential insights into amyloid research and proposes a novel peptide for advancements in materials science. In this regard, in silico studies were performed to explore the FEYNF peptide’s ability to form polyplexes.

## 1. Introduction

In recent years, the spotlight in the realms of biology, chemistry, and medicine has intensified on short peptides, which have captivated the scientific community with their distinctive features [1,2]. Apart from their low molecular weight and facile synthesis, these types of peptides are recognized for their biocompatibility and the ability to target specific cells or organelles with minimal side effects [3]. These distinct attributes make them exceptionally well suited for an extensive array of applications, such as cell culture [4], tissue regeneration processes [5], drug delivery [6], and biosensors [7]. Noteworthy is that short peptides were proven to play pivotal roles in the advanced gene therapy area [8,9]. Peptides can serve as smart delivery vehicles due to their capability to bind, conjugate, or encapsulate DNA or RNA [9]. Additionally, they are utilized in diagnostic imaging due to their inherent targeting ability [10,11].

Self-assembly represents an innate and intricate mechanism observed across numerous essential biological processes, underscoring its fundamental role in nature [12]. Within this context, peptides demonstrate a remarkable capability to autonomously organize and undergo self-assembly, particularly under specific environmental conditions [1,2,3,4]. The molecular self-assembly process implies the formation of physical hydrogels, also known as supramolecular hydrogels [13]. The predominant mechanism in peptide supramolecular gelation is primarily catalyzed by fundamental physical forces, such as hydrogen bonding, hydrophobic interactions, aromatic π–π stacking interactions, and electrostatic interactions, which are weak and reversible [14]. Such noncovalent interactions are known for imparting order and directionality to peptide self-assembly into fibril-like structures and hence play a critical role in building the final 3D architecture [15,16]. The inherent biocompatibility, high water content, and exceptional hydrogelation propensity brought on by the unidirectional amide H-bonding and π–π stacking interactions can provide peptide-based hydrogels significant unique properties [17]. These special features, when harmonized with the inherent bioactivity intrinsic to the peptide sequences, pave the way for the creation of distinct and specialized biomaterials.

It was previously reported that peptides consisting of fewer than eight amino acids can spontaneously self-assemble into hydrogels [18]. The ability of peptides consisting of only two amino acids (shortest self-assembling motif of peptides) to form linear dipeptide hydrogels was also shown, employing different synthesis strategies [13]. Chemical synthesis of peptides can be achieved either by solution- or solid-phase strategies. However, the solid-phase peptide synthesis (SPPS) method (in which a peptide is anchored to an insoluble solid support, typically made of a polymer matrix), has lately seen consistent advancements and refinements in order to produce peptides with high purity [19]. Thus, the exploration of amino acids and their ability to form specific sequences and implicitly supramolecular structures have paved the way for pioneering advancements in materials science [20,21].

Due to the intriguing structures formed by short hybrid peptides, there is growing interest in leveraging self-assembly principles to design and forecast peptide hydrogels. Therefore, understanding the underlying mechanisms and microscopic behavior involved in peptide self-assembly is essential. Computational techniques have increasingly become instrumental in elucidating the interplay between molecular structures, as well as the potential for supramolecular gelation [22]. Furthermore, the computational methodologies are expected to be critical for researchers exploring and innovating in the realm of peptide hydrogel materials.

Therefore, this research aims to explore the synergistic relationship between the synthesis of a new short-peptide, its self-assembly capacity, and its potential therapeutic applications. Accordingly, the synthesis of a new highly pure amidated pentapeptide was envisaged, specifically, FEYNF-NH_2_, comprised of F (phenylalanyl), E (glutamyl), Y (tyrosyl), and N (asparaginyl) residues. In addition to examining the morphostructural properties of the peptide, this study emphasized the significance of computational techniques, notably molecular docking simulations, to: (i) undertake an in-depth investigation into the driving forces and structural intricacies driving the self-assembly of the FEYNF peptide; (ii) forecast its inclination towards adopting a β-sheet structure and potential for supramolecular gel formation; and (iii) conduct a computational assessment (in silico study) to evaluate the FEYNF peptide’s capability to form polyplexes, serving as an initial step toward exploring potential future applications.

## 2. Results and Discussion

### 2.1. Solid-Phase Synthesis of Novel FEYNF-NH_2_ Pentapeptide

**Figure 1:**
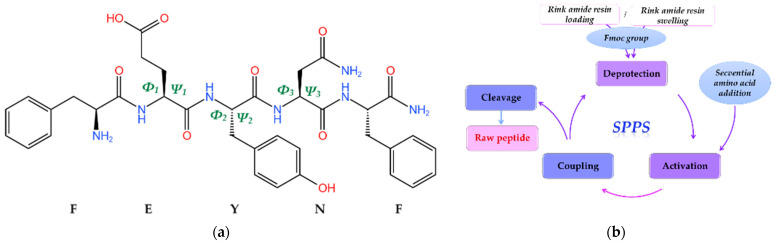
(**a**) Chemical structure depiction (2D geometry) of the FEYNF-NH_2_ pentapeptide (KingDraw V3.0.2.) highlighting *Φ* (Phi) and *Ψ* (Psi) torsion angles; where *Φ =* C(*j* − 1) − N(*j*) − Cα(*j*) − C(*j*) and *Ψ =* N(*j*) − Cα(*j*) − C(*j*) − N(*j* + 1); *j*—atom index in the backbone sequence; (**b**) schematic representation of the solid-phase peptide synthesis (SPPS) protocol (adapted from [19]).

Certain peptides have the inherent capability to undergo self-assembly, leading to the formation of gels characterized by adjustable properties under specific environmental conditions [12]. In a previous study, we demonstrated the propensity of a pentapeptide inspired by the hen egg-white lysozyme, specifically, FESNF (where F-phenylalanyl, E-glutamyl, S-seryl, and N-asparaginyl), to self-assemble into ordered and dense fibrils [23]. Given the fact that aromatic amino acid side chains (e.g., phenylalanine, tyrosine, tryptophan, and histidine) are likely to participate in π-stacking interactions [24], we suggested substituting the serine (S) in the FESNF peptide with the aromatic amino acid tyrosine (Y). This led to the synthesis of a novel amidated peptide, FEYNF, with the chemical structure (2D geometry) represented in Figure 1a.

The synthesis of the FEYNF-NH_2_ pentapeptide was effectively achieved using the solid-phase peptide synthesis (SPPS) approach, rooted in the established Fmoc/tBu methodology [15,16,25,26]. As illustrated in Figure 1b, the SPPS technique primarily entailed employing a Rink amide resin to optimize the peptide synthesis environment, along with Fmoc group deprotection, coupling of activated Fmoc–amino acids, and subsequent detachment of the peptide from the resin. It is noteworthy that amino acids with suitable protective groups were selected to ensure alignment with the 9-fluorenylmethoxycarbonyl/tert-butyl (Fmoc/t-butyl) approach for solid-phase peptide synthesis (SPPS). In addition, the amidation at the peptide’s C-terminal is expected to diminish its net charge, potentially resulting in reduced solubility. Nevertheless, this alteration augments the peptide’s stability, aligning it more closely with native proteins, thereby enhancing its biological relevance [27].

### 2.2. Qualitative FEYNF-NH_2_ Peptide Analysis and Purification

**Figure 2:**
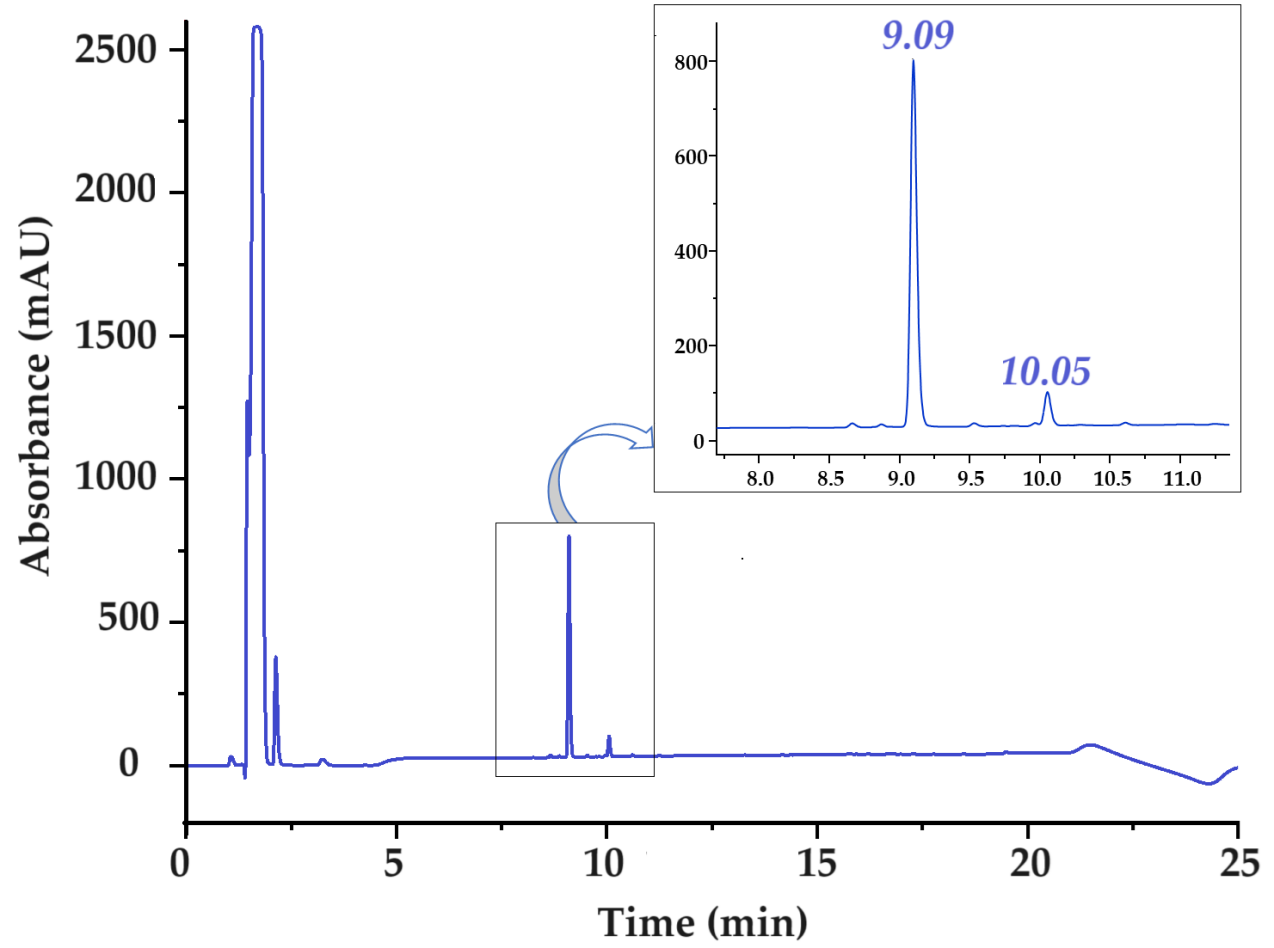
HPLC chromatogram of raw FEYNF-NH_2_ pentapeptide. The chromatogram was recorded at a wavelength of 215 nm. Injected volume: 90 µL.

Reverse-phase high-performance liquid chromatography (RP-HPLC) was used to analyze and separate the crude mixture of synthesized peptide. The HPLC profile of the raw peptide mixture FEYNF-NH_2_ is given in Figure 2. To detect the peptide bond specifically, a targeted wavelength of 215 nm was used during the analysis. The initial separation of the raw peptide mixture followed a clearly defined pattern guided by a linear gradient, as clearly depicted in Figure S1 (in Supplementary Materials). This approach facilitated the systematic fractionation of components within the mixture, allowing for discernible identification and characterization of individual compounds based on their distinct retention times.

The chromatographic analysis within the initial 5 min window revealed the presence of peaks attributed primarily to acetic acid and potential residual solvents employed during the synthesis process. However, a distinct compound, detected at 9.09 min of retention time, was unequivocally identified via mass spectrometry as the FEYNF peptide. The considerable intensity of this peak strongly indicates a successful synthesis process. Notably, a secondary peak appearing at 10.05 min of retention time, albeit with lower intensity, corresponds to a synthesis by-product demonstrating heightened hydrophobicity. This phenomenon, characterized by a marginally extended elution time, is a commonly observed occurrence in solid-phase peptide synthesis (SPPS) using Rink amide as solid support [28].

In this investigation, we employed a purification methodology for the FEYNF peptide via RP-SPE chromatography with gradient elution, a technique adapted from [29]. Notably, our protocol differed by introducing a crucial modification: after peptide elution with acetic acid, the mixture underwent adjustment to an optimal intermediate pH of 4.5, in accordance with the column cartridge specifications. This adjustment, within the pH range of 2–8 to prevent denaturation of the stationary phase, served a dual purpose: ensuring the integrity of the stationary phase and eliminating the need for a time-consuming freeze-drying step typically required to remove excess acetic acid. By preventing denaturation and obviating extended drying procedures, this adaptation streamlined our purification process, significantly enhancing efficiency and expediting the overall workflow.

It should be mentioned that the determined purity of the pentapeptide FEYNF-NH_2_ was assessed immediately after synthesis (raw product) and quantified at an impressive 95.47%. Moreover, the compounds eluted within the initial 5 min of the chromatography predominantly stem from the presence of acetic acid used for solubilization/during the washing step and residual solvents originating from the synthesis process, as well as the injection peak [30]. To guarantee the utmost quality of the peptide, a meticulous purification step was executed through solid-phase extraction. This rigorous process yielded a confirmed purity of 99.54% for the pentapeptide used in the experiments. Importantly, this achieved purity underwent thorough validation through subsequent HPLC analysis, as illustrated in Figure S2 (in Supplementary Materials).

### 2.3. Molecular Mass Confirmation (MALDI-ToF MS and MS/MS)

**Figure 3:**
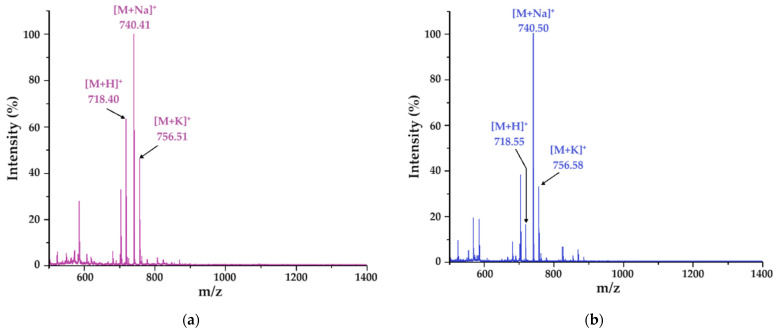

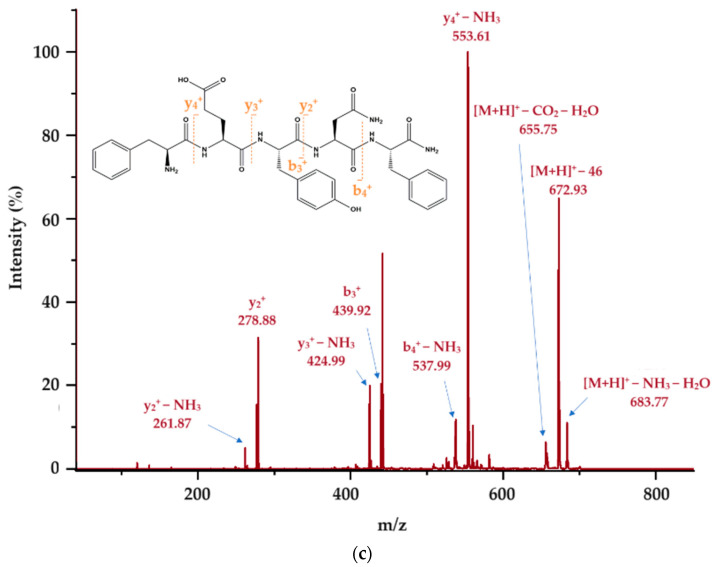
MS spectrum of the eluted fraction at 9.09 min confirming *m*/*z* corresponding to FEYNF-NH_2_ with two different matrices: (**a**) DHB and (**b**) HCCA; (**c**) assignment of *m*/*z* signals resulting from MALDI-ToF MS/MS tandem mass spectrometry analysis; fragmentation of the molecular ion [FEYNF + H]^+^ (*m*/*z* 718.55) was performed by collision-induced dissociation (CID).

Molecular mass determination was obtained by using a MALDI-ToF MS instrument (Bruker, Billerica, MA, USA). Crystals were characterized by distinctive morphologies when the peptide was associated (co-crystalized) with HCCA and DHB matrices, as depicted in Figure S3 (in Supplementary Material) and observed through the MALDI-ToF camera. Consistent with both experimental findings and existing literature, co-crystals originating from the DHB matrix exhibit a needle-like, acicular structure, while those formed from the HCCA matrix display a compact, amorphous appearance [31]. Upon mass spectrometry analysis of the first peak, the molecular ion [M + H]^+^ of the pentapeptide was identified at *m*/*z* 718.40 with the DHB matrix (Figure 3a) and slightly shifted to *m*/*z* 718.55 with HCCA (Figure 3b). Notably, alongside the protonated molecular ion, the detection of sodium ([M + Na]^+^) adduct ions at *m*/*z* 740.41 and *m*/*z* 740.50, contingent upon the specific matrix employed, was evident. The peptide appears to demonstrate a distinct tendency to produce sodium ions, possibly due to interactions between the sodium ion and carboxyl oxygen and/or amide nitrogen. This interplay could achieve stability through the aromatic ring of the tyrosine side chain, a hypothesis previously supported by previous research studies [32]. Furthermore, potassium species ([M + K]^+^) were observed at *m*/*z* 756.51 and 756.58, as depicted in Figure 3a,b.

Further analysis involved collision-induced dissociation (CID) of the molecular ion [FEYNF + H]^+^ (*m*/*z* 718.55), yielding multiple b^+^ and y^+^ molecular fragments, providing valuable insights into the fragmentation patterns of the FEYNF-NH_2_ pentapeptide and enhancing our understanding of its structural characteristics (Figure 3c). The tandem mass spectrum exhibits several distinct fragments, notably featuring ions abundant due to the elimination of ammonia: y_2_^+^-NH_3_ at *m*/*z* 261.87, y_3_^+^-NH_3_ at *m*/*z* 424.99, b_4_^+^-NH_3_ at *m*/*z* 537.99, and y_4_^+^-NH_3_ at *m*/*z* 553.61. Additionally, water loss correlates with the glutamate moiety, while carbon dioxide release likely originates from the decarboxylation of the same moiety.

Notably, at *m*/*z* 672.93 ([M + H]^+^—46), the observed 46 Da discrepancy is postulated to correspond to the elimination of a CO_2_ and a H_2_ molecule. Moreover, scrutiny of the tandem MS spectra reveals that the equitable distribution of aromatic residues along the peptide backbone induces fragmentation at all amide bonds. This diverges from our earlier investigations, wherein the mutant peptide FESNY-NH_2_ characterized by aromatic residues solely at the termini exhibited a marked predilection for fragmentation in those regions [33]. The inclusion of phenylalanine and tyrosine residues, endowed with a relatively high electron density, is conjectured to facilitate fragmentation by promoting charge localization and augmenting susceptibility to dissociation processes in tandem MS spectra [34].

### 2.4. Self-Aggregation of FEYNF-NH_2_ Investigations

#### 2.4.1. Fourier-Transform Infrared Spectroscopy (FTIR)

The investigation into peptide self-assembly and its correlation with secondary structure, particularly the formation of β-sheet structures, involved the use of FTIR spectroscopy analysis. To elucidate the secondary structure of the FEYNF-NH_2_ peptide, the examination focused on the amide bands, specifically, the amide I band. Figure S4 in the Supplementary Materials illustrates the broad shape of the amide I band, attributed to overlapping bands from distinct secondary structures, primarily consisting of peptide backbone C=O stretching vibrations [35]. Additionally, the amide II region (Figure S4) mainly reflects N–H and C–N bending modes [36], with the absorption band at 1511 cm^−1^ potentially linked to the tyrosine residue’s aromatic ring, according to De Meutter et al., which suggests its occurrence at higher wavenumbers in free amino acids [37].

**Figure 4:**
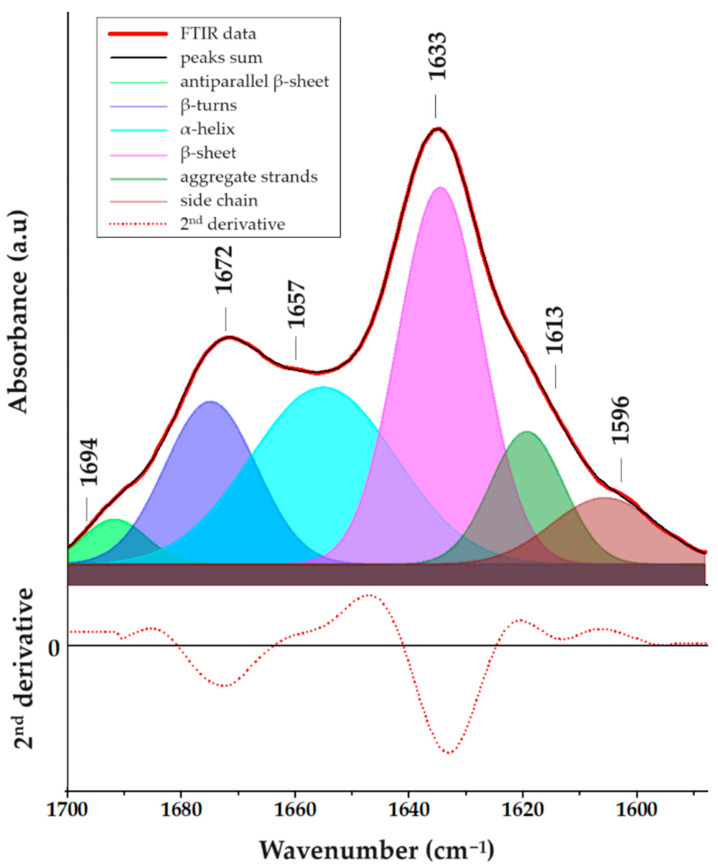
FTIR spectrum corresponding to the amide I region evidencing the secondary structures of FEYNF-NH_2_ peptide in the solid state, achieved through deconvolution (Gaussian curves positioned at peaks predetermined by the second derivative spectrum of the initial data).

For a detailed assessment of the FEYNF-NH_2_ peptide’s secondary structure, quantitative Gaussian peak fitting was applied to the FTIR data (Figure 4), utilizing OriginPro 8.5.0 SR1 software (OriginLab Corporation, Northhampton, MA, USA). Following a linear baseline subtraction for the amide I band (Figure S4), the second derivative was calculated to identify the position and number of absorption bands essential for peak fitting, employing a 20-point Savitzky–Golay smoothing function [38]. Subsequently, Gaussian peaks were introduced into the amide I band based on positions indicated by the second derivative spectrum, and the corresponding structures were identified referencing literature [35,36,37,38], as shown in Figure 4.

One may see that most notable absorption bands are located at 1633 cm^−1^ and 1672 cm^−1^, corresponding to β-sheet and β-turn structures, respectively. This observation is supported by the data presented in Table S1 from the Supplementary Materials, where it is revealed that the peaks associated with β-sheet/turn structures contribute significantly to the overall area of the amide I band. However, while the self-assembly of FEYNF-NH_2_ peptide is primarily driven by the formation of β-sheet structures, it is noteworthy that FTIR spectrum may also exhibit signals indicative of α-helix and side chain structures. Nevertheless, as indicated by FTIR analysis, the primary structural pattern observed in the self-assembly of peptides is linked to the formation of β-sheets. As a result, further analysis will be employed to deepen and understand the intricacies of FEYNF-NH_2_ peptide self-assembly.

#### 2.4.2. Fluorescence Studies

**Figure 5:**
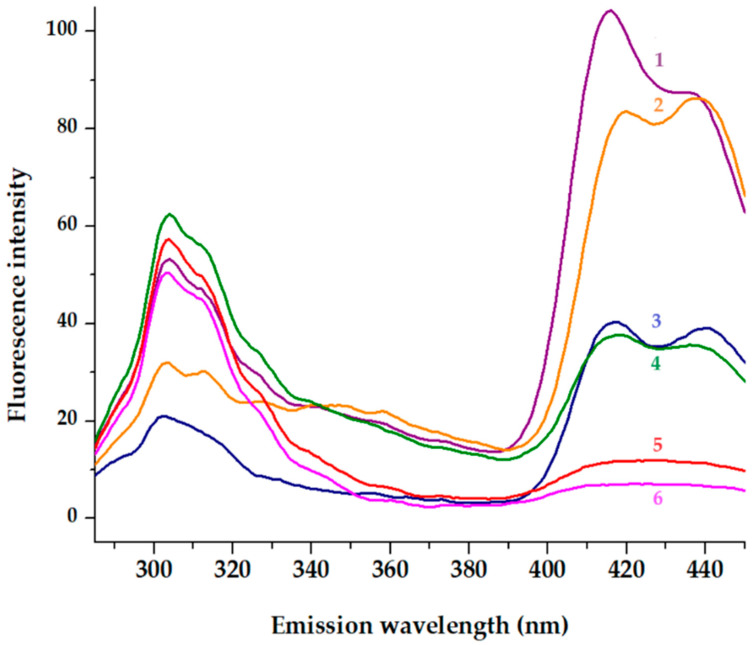
Fluorescence emission spectra recorded at λ_ex_ = 275 nm. The initial spectra (1, 4) of peptide at 27 µM respectively 0.5 mM are displayed. The emission spectra of peptide after 10 min (2 and 5) and 20 min (3 and 6) incubation at room temperature (20 degrees) are presented.

The fluorescence studies conducted in sodium acetate at pH 7.4, along with the concentration-dependent experiments, provide essential contributions to understanding the dynamics of self-assembly. To universally excite the two aromatic amino acids, phenylalanine and tyrosine, embedded within the peptide backbone, an excitation wavelength of 275 nm was employed [39]. As represented in Figure 5, the noticeable decline in fluorescence intensity at 304 nm, coupled with the concurrent decrease in the shoulder within the 322–335 nm range after 20 min, alongside a reduction in fluorescence intensity in the violet-blue region (390–450 nm), collectively signifies significant changes in the microenvironment enveloping the aromatic amino acids (phenylalanine and tyrosine). These observed alterations strongly suggest a compelling correlation with the gelation process [40]. Furthermore, the dityrosine formation characterized by a emission signal at around 409 nm cannot be excluded [41].

Expanding the investigation to include the violet-blue region is particularly insightful. The violet-blue region encompasses emissions from both phenylalanine and tyrosine, revealing additional nuances in their fluorescence behavior. This broader spectral analysis allows discerning changes in the electronic environment, potential shifts in molecular conformations, or alterations in the interactions among aromatic amino acids during gelation. The reduction in fluorescence intensity in the violet-blue region suggests a complex interplay of factors affecting the electronic states of both phenylalanine and tyrosine. This could include changes in solvent accessibility, local structural rearrangements, or variations in intermolecular interactions, all of which contribute to the observed fluorescence behavior. By considering these additional spectral features, the understanding of the molecular events associated with gelation becomes more nuanced and provides valuable insights into the intricate dynamics of the peptide’s structural transformation.

This quenching phenomenon was observed within a brief timeframe for two distinct concentrations of the FEYNF-NH_2_ peptide: 27 µM and 0.5 mM (Figure 5). The quenching effect in fluorescence, observed in the blue region of emission spectra, can be attributed to a multitude of factors, encompassing alterations in fluorophore proximity and interactions with neighboring molecules. Particularly, in the context of gelation, the formation of a three-dimensional network is believed to foster heightened interactions among aromatic amino acids, thereby influencing their fluorescence properties [42,43]. It is well known that tyrosine quenching and a tyrosine–tyrosine homotransfer might be interconnected [44]. Further insights into these intricate dynamics can be crucial for a comprehensive understanding of the peptide’s behavior under different concentrations and temporal conditions.

#### 2.4.3. Transmission Electron Microscopy (TEM)

In both solid and solution phases, under specific conditions, peptide subunits engage in intricate noncovalent interactions, leading to the formation of self-assembled architectures. These supramolecular constructs subsequently undergo further self-association, culminating in diverse nano- and micro-architectures pivotal for the advancement of functional materials [45]. Consequently, to evaluate the morphological features of the newly synthesized FEYNF-NH_2_ peptide, transmission electron microscopy (TEM) was employed (Figure S5a,b, Supplementary Material). In addition, the elemental composition and the purity of the synthetized peptide were confirmed by the presence of characteristic C, N, and O elements (see Figure S5c,d in Supplementary Materials), using energy-dispersive X-ray spectrometry (EDX) coupled with TEM. It should be noted that the differences in C intensities are mainly due to the use of TEM carbon grids.

The TEM images of the pentapeptide (previously dispersed in phosphate buffer solution and dimethyl sulfoxide (DMSO)) are depicted in Figure S5a (in Supplementary Material). One may see the self-assembled peptide as a highly extended nanotape with overlapping areas. This could be attributed to an increase in the density of transitory noncovalent contacts between self-assembled nanostructures [46]. In addition, this arrangement (flat, extended shape) is characteristic of β-sheets. The corresponding TEM image for the dehydrated peptide after incubation is delineated in Figure S5b (Supplementary Materials). In contrast to Figure S5a, the incubation of the FEYNF peptide appears to instigate the development of structures resembling fibrils, along with some regions exhibiting tape-like characteristics.

#### 2.4.4. Polarized Optical Light Microscopy (POM)

**Figure 6:**
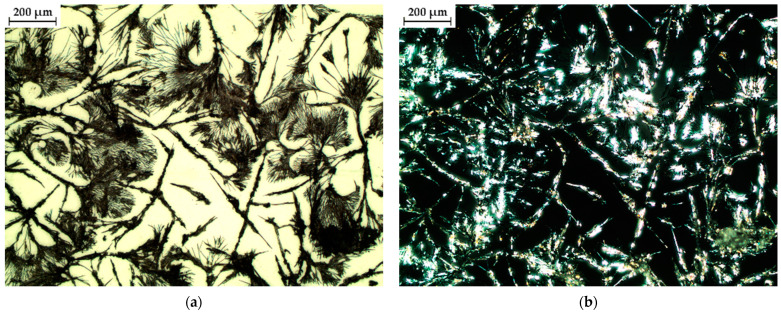

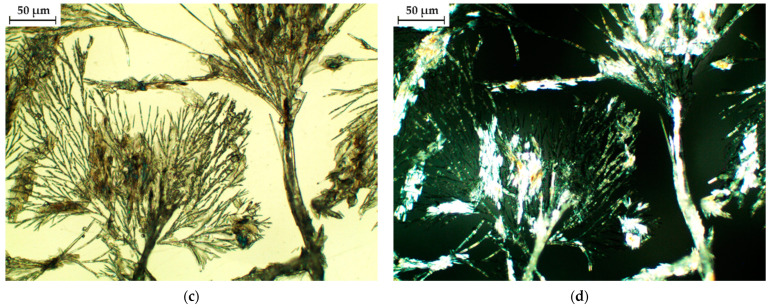
Microscopic images of dehydrated specimens derived from incubated solutions (3 mM) of FEYNF-NH_2_ at a physiological pH of 7.4 and a temperature of 37 °C, captured under two polarization angles: 0 degrees (**a**,**c**), and 90 degrees (**b**,**d**) and at two different scales (50 and 200 μm).

A major factor determining the physical and chemical properties of self-assembled peptides (and amino acids) is molecular orientation [21]. Polarization-based optical analysis is very useful in molecular analysis, mainly when it comes to molecular orientation. Thus, in order to have a more in-depth perspective on the morphology of the peptide after incubation, polarized light microscopy was used. Accordingly, in Figure 6, the images obtained at two different polarizing angles (0° and 90°) and also at two different magnifications are represented. One may see that the self-assembled FEYNF peptide reveals intricate hyperbranched architecture, characterized by expansive dendritic patterns. Figure 6c highlights the increased visibility of short branches that have formed and are oriented nearly perpendicular to the nanofiber axis. This behavior was reported by Chakraborty et al., revealing the presence of entangled fibers following gelation, a characteristic of supramolecular hydrogels [17].

The FEYNF morphology can be mainly attributed to the presence of a tyrosine amino acid residue. It is well known that within physiological parameters (pH 7.4), the tyrosine residue likely retains its neutral state, facilitating pivotal interactions for the initiation of fibrillogenesis [47]. When juxtaposed with the previously studied peptide (FESNF-NH_2_) under analogous pH conditions [23], the introduction of tyrosine led to morphological changes for FEYNF-NH_2_. Hence, although the formation of fibrils was absolutely absent in the case of the initial FESNF-NH_2_ peptide at pH 7.4, and the newly synthetized FEYNF-NH_2_ peptide possesses a distinctive capability at the physiological pH: the formation of dendritic-like fibrils (Figure 6). This structural variation predominantly stems from the tyrosine’s aromatic ring and the presence of an adjacent hydroxyl moiety. Thus, in addition to the Phe–Phe interaction (previously shown to ensure π–π stacking and hydrophobic interactions), tyrosine may similarly contribute to peptide self-assembly. Interestingly, the theoretical calculated isoelectric point of the FEYNF peptide (6.94) closely aligns with the native FESNF peptide isoelectric point (6.99). Nonetheless, FEYNF-NH_2_ exhibits resemblances in fibril formation with the amyloid-beta peptide (Aβ), especially when contextualized within the vicinity of its isoelectric point [48]. This noteworthy characteristic not only holds potential for designing related compounds but also offers fresh insights into the role of amino acids in triggering the emergence of gel aggregates.

An interesting aspect is visible in Figure 6b,d registered at a polarization angle of 90°. Under polarized light, the self-assembled FEYNF peptide exhibits birefringence. This indicates the self-assembly governed by the presence of peptide β-sheets, which led to well-organized, crystalline structures. It has been shown by others that temperature can also influence peptide self-assembly, resulting in bundles of aligned peptide nanofibers. These bundles can produce macroscopic birefringent domains on the order of tenths of millimeters [21,49].

To further investigate using polarized light microscopy, we evaluated the Congo red staining assay. Congo red is acknowledged for its affinity to bind with β-sheets and adhere to amyloid structures, characterized by either complete or partial composition of β-folded sheets. In the context of this study, Lembré et al. observed that amyloid peptides tend to exhibit a distinctive “apple-green” birefringence upon Congo red staining when examined under polarized light [50]. However, the perceived dichroism in amyloid structures, particularly the apple-green color, is considered potentially misleading as it is specific to stained fibrils perfectly aligned with the polarized light beam. Minor deviations, such as changes in the polarized light angle, can result in the manifestation of “anomalous” colors [51]. Our findings align with this perspective, as the microscopic images of Congo red-stained FEYNF-NH_2_ peptide were captured under different incidence angles (0°—Figure S6a and 90°—Figure S6b). Thus, comparison with Figure S6a,b indicates the presence of dichroic birefringence. This supports the existence of peptide β-sheets, essential in governing the self-assembly of the peptide.

### 2.5. Molecular Docking Simulation

The process of self-assembled supramolecular gelation operates across multiple scales, with atomic-scale interactions dictating the properties of macroscopic hydrogel materials. As a result, a variety of computational methods with varying levels of precision are essential to gathering information at distinct scales [22]. Commonly employed within computational simulations, the molecular docking technique is extensively utilized for scrutinizing interactions between a ligand, typically an organic molecule, and a receptor, such as oligomers, proteins, DNA, RNA, or other macromolecules. More precisely, this in silico method enables: (i) the anticipation of the ligand’s spatial arrangement within a binding site, commonly known as its “pose”; (ii) the evaluation of the binding affinity in the ligand–receptor complex through two parameters, namely, binding energy and dissociation constants; and (iii) the identification of the interactions likely to occur between the ligand and the receptor [52]. The AutoDock-VINA algorithm (included in the YASARA Structure v.20.8.23 program) is more like a “machine-learning” approach, amalgamating the merits of knowledge-based potentials and empirical scoring functions [53,54,55]. This approach is employed to evaluate the binding energy associated with a specific ligand pose within the binding site of the receptor.

#### 2.5.1. Insights into FEYNF-NH_2_ Self-Assembling Mechanism

**Figure 7:**
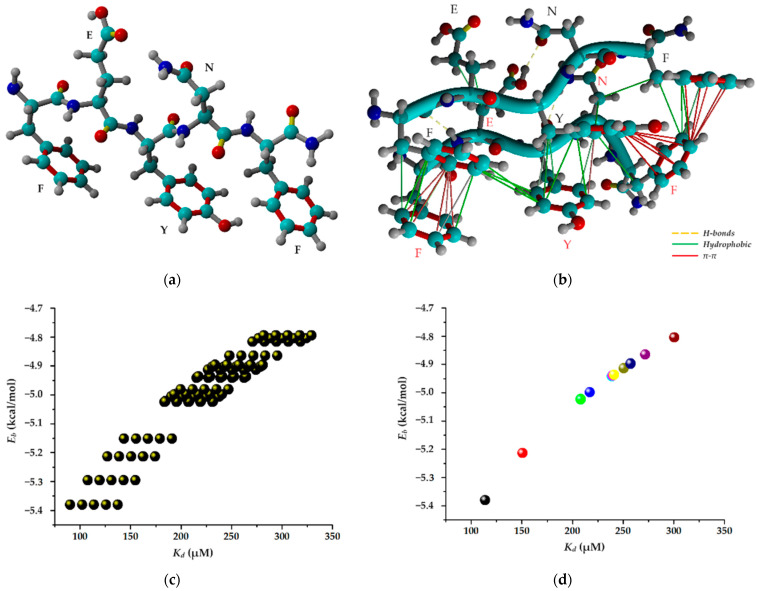
(**a**) Optimized conformer (3D structure) of FEYNF-NH_2_ pentapeptide at the level of YASARA force field (atom colors: cyan—carbon, blue—nitrogen, red—oxygen, and gray—hydrogen); (**b**) best docked FEYNF@FEYNF dimer complex (peptide ligand–black annotations and peptide receptor–red annotations); (**c**) data for all 100 docked poses of FEYNF@FEYNF dimer complex demonstrating the relationship between binding energy and dissociation constant; (**d**) data for clustering analysis for the 11 representative docked models (clusters).

The computer-aided molecular docking simulation was performed herein in order to gain comprehensive insights into the peculiar mechanism governing the self-assembly of the amidated FEYNF peptide. Assuming the chemical structure (2D) of the pentapeptide given in Figure 1a, its spatial conformer (3D) was built directly in the YASARA-Structure program using the “BuildMol” command. Subsequently, the initial generated 3D geometry of FEYNF-NH_2_ underwent optimization using the molecular mechanics method at the YASARA force field level [56]. The optimized 3D arrangement of the atoms in the FEYNF-NH_2_ structure (following energy minimization) is illustrated in Figure 7a. One may observe that the hydrophilic and hydrophobic peptide side chains are found on opposite sides of the peptide backbone. The optimized conformation from Figure 7a was then utilized in molecular docking simulations, serving dual roles as both a flexible ligand and a rigid receptor, to spot the self-assembling mechanism by assessing the peptide–peptide interactions during the dimer formation (Figure 7b).

Molecular docking simulations were carried out by assessing 100 docked complexes (poses) of the dimer (FEYNF@FEYNF), using the VINA algorithm. The numerical data obtained for all 100 poses (plotted in Figure 7c) elucidate the correlation between binding energy (*E_b_*) and dissociation constant (*K_d_*) for each docked model. As shown in Figure 7c, an increased value of the dissociation constant for a given docked model corresponds to a higher associated binding energy. Nevertheless, the relationship (*K_d_* vs. *E_b_*) is non-linear and characterized by a monotonically increasing trend. After clustering the 100 peptide dimer conformations, 11 distinct and representative docked models (clusters) were identified. The clusters differ from each other in terms of root-mean-square deviation (RMSD) of atomic positions of at least 5.0 Å. Subsequently, Table S2 (in Supplementary Materials) provides detailed information on binding energy (*E_b_*), dissociation constant (*K_d_*) values, and the amino acid residues involved in the peptide–peptide interaction for each cluster. The relation between the binding energy (*E_b_*) and dissociation constant (*K_d_*) after the clustering analysis is represented in Figure 7d. Generally, the lower the values for *E_b_* and *K_d_*, the better the affinity, and the peptide–peptide interaction is more favorable, indicating a higher docking score. Consequently, according to Figure 7d and Table S2 (Supplementary Materials) data, the optimum pose for the peptide dimer complex was associated with the lowest binding energy (*E_b_* = −5.38 kcal/mol) and dissociation constant (*K_d_* = 113.87 μM). The calculated values indicate a strong and stable noncovalent binding interaction between the peptide molecules. This can be attributed to the fact that each distinctive amino acid residue from the FEYNS-NH_2_ structure (PHE1 (F), GLU2 (E), TYR3 (Y), ASN4 (N), and PHE5 (F)) is involved in the peptide–peptide interaction, as indicated by the data in Table S2.

Hence, the best docked FEYNF@FEYNF dimer complex conformation and the corresponding binding interactions are highlighted in Figure 7b. One may see that the peptide structures are oriented in the same direction (parallel), and the intermolecular interactions between the peptide molecules are based on hydrophobic interactions, π–π stacking, and H-bonds (detailed in Table S3, Supplementary Materials). The presence of multiple interacting sites is ideal for the formation of supramolecular gels.

Appropriate intermolecular alignment and interactions between side chains are required for a certain peptide conformation with the assembly of nanofibers into a hydrogel. As anticipated, in concordance with findings from a previous investigation [23], the presence of terminal PHE side chains (PHE1 and PHE5) in the peptide’s structure facilitates their engagement in π–π stacking interactions. Such interactions are recognized for imparting order and directionality to the peptide self-assembly into fibril-like structures, playing a pivotal role in shaping the final 3D architecture [23,57]. However, in the present study, the introduction of the TYR3 (tyrosyl, Y) moiety into the peptide structure resulted in additional π–π stacking interactions with the PHE5 terminal residue (Figure 7b and Table S3). Thus, in addition to the available PHE terminal sequences, which promote linear aggregation via the hydrophobic effect, the TYR3 sequence offers the possibility of branched growth. This can explain the self-assembling of the FEYNF-NH_2_ peptide into a hydrogelated dendritic architecture, as evidenced above in Figure 6 (under polarized light).

#### 2.5.2. Investigation of β-Sheet Structure Formation

**Figure 8:**
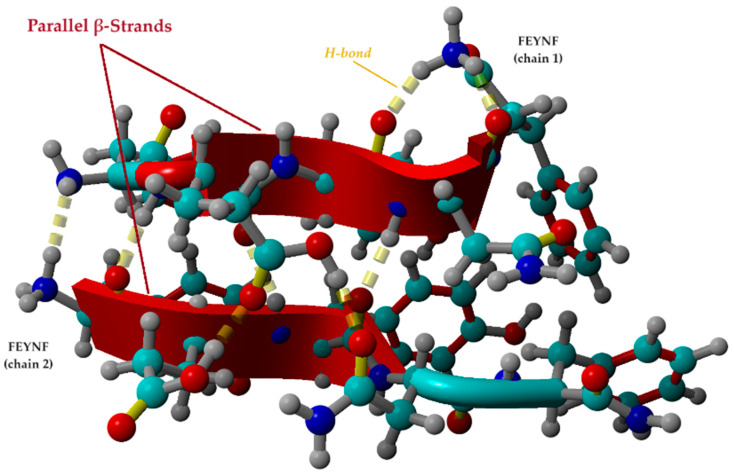
Optimal conformation of the protonated dimmer (FEYNF@FEYNF(+2)) computed at the level of the YASARA force field, suggesting the self-assembling and gelation pathway of FEYNF toward an β-sheet structure (atom colors: cyan—carbon, blue—nitrogen, red—oxygen, and gray—hydrogen).

It is important to note that one important criterion for determining whether peptides can self-assemble into hydrogelated structures is their tendency toward a β-sheet conformation [22]. Given the fact that the optimal conformation of the FEYNF peptide (Figure 7a) revealed the distribution of the hydrophilic and hydrophobic side chains on the opposite sides of the peptide backbone, it is expected that β-strand formation will occur [58]. In this context, the best pose for FEYNF@FEYNF dimer complex was further investigated considering its protonated form (-NH^3+^). Thus, the modeled peptide–peptide complex carried a net charge equal to (+2) by protonation of one amine group for each peptide chain at the opposite end. The molecular conformation of the protonated dimer complex was then subjected to energy minimization by molecular mechanics theory at the level of the YASARA force field. Consequently, the optimal conformation of the modeled protonated dimer (FEYNF@FEYNF(+2)) is depicted in Figure 8.

Hence, following energy minimization, the tertiary structure of the peptides forms two β-strands with the same N to C orientation (parallel configuration). The β-strands given in Figure 8 are mainly formed by the backbone in an extended conformation of the GLU2, TYR3, and ASN4 for chain 1 of the FEYNF peptide. For the second chain, the β-strand starts from the C-(C=O) group of the PHE1 and continues with the GLU2 and TYR3 backbone.

In the depicted conformation (Figure 8), the amidated FEYNF peptide exhibits the capacity to assume a β-sheet structure, as the two represented β-strands are laterally connected by H-bonds. This phenomenon facilitates the extension of β-sheets with an increase in size, particularly as the oligomer expands, inherently implying the formation of a fibril. To confirm the β-sheet formation in the dimer conformation optimized by molecular mechanics at the level of the YASARA force field (Figure 8), the dihedral (torsion) angles *Φ* (Phi) and *Ψ* (Psi) were calculated for each chain, and their values are given in Table 1. The *Φ* torsion angle is characteristic for the N − Cα bond, while the *Ψ* angle corresponds to Cα − C bond (as shown in Figure 1a), both types of bonds being flexible and free to rotate. The values for the torsion angles can lead to the determination of the 3D shape of the polypeptide backbone, in accordance with the Ramachandran plot [59].

The torsion angles, as outlined in Table 1, validate the existence of an elongated conformation conducive to β-sheet formation. The pair values (*Φ*,*Ψ*), depicted in Figure S7 (in Supplementary Materials), align with the β-sheet region of the Ramachandran plot [59]. An exception is observed in the case of the pair (*Φ*_3_,*Ψ*_3_) for the FEYNF chain 2. This is attributed to the absence of the ASN4 residue in the β-strand formed within the FEYNF chain 2 (as also observed in Figure 8). However, the amidated FEYNF peptide sequences’ β-strand conformation confirm their ability to engage in the self-assembly process, generating hydrogelated architectures with a β-sheet tertiary structure.

### 2.6. Potential Use of FEYNF Self-Assembling Aggregates in Polyplex Formation

Polyplexes are a special type of macromolecular complex that have garnered significant attention in recent decades owing to their potential applications in gene therapy. These specialized complexes arise through the interaction of (macro)molecular entities, such as polypeptides and polycations, with nucleic acids (DNA or RNA) [60]. Gene delivery systems are currently divided into two primary directions: viral and non-viral vector systems. Given the substantial risks associated with viral vectors, increased focus has shifted toward non-viral systems, predominantly relying on the use of cationic lipids, cationic polymers, or cationic polypeptides [61]. For example, it was demonstrated the efficiency of dendritic amino acid-conjugated polyamidoamines as carriers for human adipose-derived mesenchymal stem cells (AD-MSCs), as they exhibit lower immunogenicity and cytotoxicity, as well as higher transfection efficiency [62]. Peptide-based non-viral vectors are also considered ideal candidates for gene therapy, mainly due to their cell-penetrating capacity [63]. In this context, the polyplex formation capacity of the FEYNF peptide was investigated by molecular docking simulations (in silico study).

Hence, the optimized protonated FEYNF@FEYNF dimer complex (shown in Figure 8) was employed in a second molecular docking simulation study using a DNA oligonucleotide sequence as the receptor. To this end, the Drew–Dickerson dodecamer d(CGCGAATTCGCG)2 was used as a model for the short DNA helix simulation [60,64]. Details regarding the molecular structure of the dodecamer are given in Figure S8 (in Supplementary Materials). Hence, the protonated peptide dimer (FEYNF@FEYNF(+2)) was modeled as a rigid ligand, while the negatively charged Drew–Dickerson dodecamer (DDD (−25)) was modeled as a rigid receptor. In this instance, after running 100 poses for the polyplex conformation, 12 representative docked models (clusters) were identified. The results of the molecular docking calculations, encompassing information on binding energy, dissociation constant, and contact residues, are comprehensively presented in the Supplementary Material (Table S4 and Figure S9).

**Figure 9:**
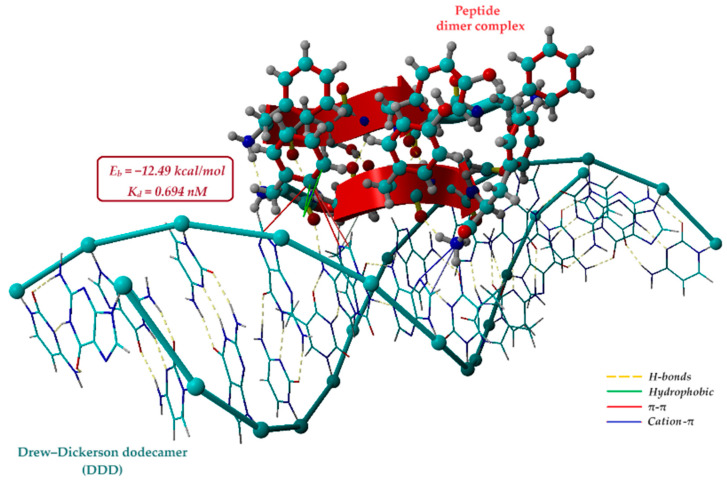
Best conformation of the polyplex resulting from molecular docking simulation showing the noncovalent interactions between the protonated dimer (FEYNF@FEYNF) as ligand and the Drew–Dickerson dodecamer d(CGCGAATTCGCG)2 as receptor (atom colors: cyan—carbon, blue—nitrogen, red—oxygen, and gray—hydrogen).

The optimal docking pose between the protonated dimer complex (FEYNF@FEYNF(+2)) and the DNA oligonucleotide sequence (DDD (−25)) is shown in Figure 9. Consequently, the most favorable affinity was observed at the lowest binding energy (*E_b_* = −12.49 kcal/mol) and the smallest dissociation constant (*K_d_* = 0.694 nM). Notably, the nanometric order of the dissociation constant value indicates the formation of a highly stable docked complex, affirming the successful formation of the polyplex. As illustrated in Figure 9, polyplex formation relies on noncovalent interactions, such as hydrophobic interactions, inter- and intramolecular H-bonds, π–π stacking, and cation–π interactions. The attractive π–π interactions primarily occur between the π bonds of the PHE1 aromatic ring in the peptide dimer and the fused-ring molecules of the DNA purine nucleobases (G4-guanine and A5-adenine). Additionally, Figure 9 also highlights cation–π interactions involving the positively charged amino group at PHE5 (chain 1 of the docked dimer) and the π–electron cloud of aromatic rings in adenine (A6) and thymine (T7). All these noncovalent interactions significantly contribute to the stabilization of the polyplex.

The supramolecular assembly of FEYNF peptides demonstrates the potential to compact nucleic acids, transforming B-form DNA into a more condensed sequence specific to polyplexes. This plays a pivotal role in gene therapy. This compaction process serves to safeguard nucleic acids, shielding them from premature degradation, and facilitates their transportation across the plasma membrane, particularly through cellular processes like endocytosis. Consequently, this enhances cell absorption.

## 3. Conclusions

This comprehensive multi-approach study has successfully undertaken the synthesis, characterization, and separation of a de novo peptide sequence capable of spontaneous self-assembly into unique intricately branched dendritic structures under physiological conditions. Initially, the peptide was synthesized through solid-phase peptide synthesis (SPPS) employing the Fmoc/tBu strategy and was effectively characterized using RP-HPLC and MALDI-ToF MS. FTIR analysis revealed that the FEYNF-NH_2_ peptide self-assembly is primarily driven by the formation of β-sheet structures. The fluorescence analysis showed the presence of a quenching phenomenon, observed within a brief timeframe for two distinct concentrations of the FEYNF-NH_2_ peptide. In addition, the microscopic examination through POM and TEM imaging provided valuable insights into the elaborate framework, revealing its potential for forming gel-like structures. For future research, one may consider exploring alternative methods to gain additional insights into the intricate dynamics of peptide behavior under various conditions.

The application of in silico studies employing molecular docking simulation (using the AutoDock-VINA algorithm included in YASARA-Structure) has unveiled critical insights into the peptide–peptide interactions and the conformational behavior of the protonated dimer (FEYNF@FEYNF(+2)). These computational methods have not only illuminated the self-assembling pathway but have also shed light on the intriguing gelation process. The prevalence in FEYNF to a β-sheet structure is an essential disclosure, indicating the structural evolution of the peptide and its capacity to form organized, sheet-like arrangements.

Moreover, the revelation of noncovalent interactions between the FEYNF peptide’s protonated dimer (as a ligand) and the widely acknowledged Drew–Dickerson dodecamer d(CGCGAATTCGCG)2 holds paramount importance. This DNA sequence, often employed as a key model in structural and molecular biology research, underscores the practical applicability of the FEYNF peptide. This discovery not only validates the versatility of the FEYNF peptide but also marks a significant advancement, connecting peptide self-assembly with DNA recognition. This linkage opens promising avenues for the development of innovative biomaterials and targeted therapeutics.

## 4. Materials and Methods

### 4.1. Reagents

Fmoc-L-phenylalanine, Fmoc-L-glutamic acid 5-tert-butyl ester, and N-α-Fmoc-N-ß-trityl-L-asparagine were procured from Fluka (Buchs, Switzerland), while Fmoc-O-tert-butyl-L-tyrosine was sourced from Roth (Karlsruhe, Germany). Furthermore, Rink amide resin (4-(2′,4′-dimethoxyphenyl-Fmoc-aminomethyl)phenoxy resin, PEPTIPURE^®^, 100–200 mesh, 0.3–0.8 mmol·g^−1^, 1% DVB) purchased also from Roth was used as a solid support. Various chemical reagents essential for experimental procedures were obtained from reputable suppliers. Dimethyl sulfoxide (DMSO, EMSURE ACS), diethyl ether (EMSURE ACS), N,N-dimethylformamide (DMF), piperidine (PYP), triisopropylsilane (TIS), acetic acid, benzotriazol-1-yl-oxytripyrrolidino phosphonium hexafluorophosphate (PyBOP), N-methylmorpholine (NMM), 2,5-dihydroxybenzoic acid (DHB), α-cyano-4-hydroxycinnamic acid (HCCA), and NaH_2_PO_4_ were acquired from Merck (Darmstadt, Germany). Dichloromethane (DCM) was sourced from Scharlab (Barcelona, Spain), while methanol, acetonitrile (ACN, HPLC grade), and trifluoroacetic acid (TFA) were obtained from Carl Roth (Karlsruhe, Germany). All solutions were prepared meticulously using water of high purity with a resistivity of 18.2 MΩ∙cm, generated through a Millipore Milli-Q system (Bedford, MA, USA).

### 4.2. Solid-Phase Synthesis of FEYNF-NH_2_ Pentapeptide

The amidated peptide FEYNF-NH_2_ (depicted in Figure 1a) was obtained using the solid-phase peptide synthesis (SPPS) method, employing the Fmoc/t-butyl strategy, as described in the literature [15,16,24,25]. A Rink amide resin with a coarse mesh served as the primary solid support during the synthesis. The key procedures consisted of two primary steps for coupling amino acids to the peptide chain. In the initial step, the deprotection of the Fmoc group was assessed for durations of 2, 2, 5, and 10 min using a 20% PYP in DMF solution. Subsequently, the bromophenol blue assay was used to evaluate the effectiveness of deprotection, in accordance with the methodology proposed by Krchnak et al. [65]. The second stage entailed coupling activated Fmoc–amino acids dissolved in DMF (to the exposed amino group on the resin) using PyBOP and NMM as coupling reagents. The coupling reaction lasted 50 min to guarantee the full integration of each amino acid into the emerging peptide chain. To ensure the reliability of the process, each amino acid underwent a double-coupling process. Following each deprotection phase, rigorous resin washing was conducted, involving six washes with 2 mL of DMF and an additional three washes between the first and second coupling reactions. To address time constraints, the synthesis was briefly paused by washing the resin with DCM before the next deprotection phase. Essential resin swelling was performed for 30 min in DMF before both initiating and resuming the synthesis process.

Upon completion of synthesis, a cleavage solution comprised of TFA, TIS, and deionized water (in a volumetric ratio of 18:1:1, *v*/*v*/*v*) was employed to release the peptide from the resin. This process was conducted at room temperature for 3 h under stirring. Subsequent to cleavage, the crude product was precipitated in cold diethyl ether and isolated using a fritted glass filter funnel (Porosity 4). The raw peptide fractions were then eluted using 50% and 5% aqueous acetic acid solutions, respectively.

### 4.3. Reverse-Phase High-Performance Liquid Chromatography

The raw pentapeptide underwent qualitative examination using an HPLC Dionex UltiMate 3000 UHPLC system from Thermo Fisher Scientific (Waltham, MA, USA). The non-polar stationary phase consisted of an AcclaimTM C18 column measuring 100 mm × 4.6 mm (5 µm silica, 120 Å pore size). Two eluents were used in the mobile phase: A (0.1% TFA in MilliQ water) and B (0.1% TFA in a mixture of ACN and MilliQ water, 80:20 *v*/*v*), prepared by adapting a method described by Körsgen et al. [30]. The column temperature was maintained at 25 °C throughout the entire analytical procedure.

### 4.4. Solid-Phase Extraction of FEYNF-NH_2_ Peptide

The purification of the newly synthesized FEYNF-NH_2_ peptide was accomplished through reverse-phase solid-phase extraction (RP-SPE). The purification methodology for the FEYNF peptide was RP-SPE chromatography with gradient elution, a technique adapted from Insuasty Cepeda et. al. [29]. Thus, a Waters™ Sep-Pak™ cartridge syringe (Milford, MA, USA) equipped with a hydrophobic C18 stationary phase was used [29]. A concentrated peptide fraction was eluted with 50% glacial acetic acid and adjusted to pH 4.5 with a 1 M NaOH solution. For reproducibility, the column was solvated with methanol and equilibrated with eluent a (0.1% TFA in MilliQ water). The solution containing the pentapeptide was gently loaded onto the column at a low flow rate to enhance the analyte–sorbent interaction. An additional rinsing step with MilliQ water was performed to effectively eliminate unwanted salts or residual agents. Elution involved a stepwise gradient of eluent B in eluent A ranging from 5% to 60% (see eluents described in Section 4.3). Following the assessment of purity through HPLC analysis, the eluted samples underwent freeze-drying using Christ Alpha 3–4 equipment (LSCbasic, Osterode, Germany). Consequently, the FEYNF-NH_2_ peptide was obtained as a solid.

### 4.5. Mass Spectrometry Analysis (MALDI-ToF MS and MS/MS)

The confirmation of the molecular ion was conducted using matrix-assisted laser desorption–ionization mass spectrometry (MALDI-ToF MS), employing two distinct matrices: HCCA and DHB. In this context, a Bruker Ultraflex MALDI ToF/ToF mass spectrometer was used. Peptide mapping was assessed by using a semi-saturated DHB solution (36 mg/mL) mixed with a 3:2 ratio of ACN: 0.1% TFA (in MilliQ water). In addition, a supra-saturated HCCA matrix solution was prepared in the same solvent. Following room-temperature drying, the sample was co-crystallized with each matrix in a 1:1 ratio on a 348-spot target plate. MALDI-ToF MS spectra were acquired in positive ion mode under defined conditions: 25 kV acceleration voltage, a delay of 140 ns, 50% grid voltage, and a low mass gate of 500 Da (500–1400 Da mass range). For MS calibration, a Bruker Peptide Calibration STD II containing nine reference peptides from 700 to 3500 Da was employed. Furthermore, the specific molecular ion underwent tandem mass spectrometry (MS/MS) using collision-induced dissociation (CID). The investigation of peptide fragmentation aimed to ensure precise data acquisition.

### 4.6. Fluorescence Analysis

A stock peptide solution (3 mM) was freshly prepared in Milli-Q water. Subsequently, the peptide was further diluted to two distinct final concentrations (27 µM and 0.5 mM, respectively) using a 30 mM sodium acetate solution with a pH of 7.4. Fluorescence spectra were acquired using a JASCO FP-8350 spectrofluorometer (Jasco Inc., Tokyo, Japan), with 2D spectra recorded in the emission range of 285–450 nm and an excitation wavelength of 275 nm. To ensure accuracy, each measurement was systematically repeated ten times at three distinct time intervals: initially, after 10 min, and finally, after 20 min. The peptide exhibited intrinsic fluorescence at 303 nm attributed especially to one tyrosine residue positioned in the center of the pentapeptide primary sequence. The emission could be also attributed to deep-blue autofluorescence (around 440 nm) observed prior formation of well-defined structures involving mainly phenylalanine residues positioned at the extremities and less to the tyrosyl moiety [66].

### 4.7. Sample Preparation Protocol for Incubation in Physiological Conditions

A 3 mM pentapeptide solution, formulated in a solvent mix of 10 mM phosphate buffer solution (pH 7.4) and 1% dimethyl sulfoxide (DMSO), underwent a controlled incubation at 37 °C for 20 h using the Eppendorf Thermomixer Compact (Eppendorf SE, Hamburg, Germany). Following incubation, a 5 µL aliquot of the aged solution was meticulously deposited onto a microscope slide and allowed to desiccate at room temperature. The resultant dried sample was then examined in ATR mode, by using a Bruker Vertex 70 FTIR spectrometer (Bruker, Ettlingen, Germany). In addition, a polarized optical microscope from Leica Microsystems (Wetzlar, Germany) was used for morphological analysis of the pristine peptide in solid state, as well as to observe its morphology after Congo red staining. For the latter, a 0.5% solution of Congo red was applied onto the microscope slide containing the dried peptide, followed by an additional incubation period of 30 min at 37 °C in an Orbital Shaker-Incubator ES-20/60 (Biosan, Riga, Latvia). In addition, a Hitachi High-Tech HT7700 transmission electron microscope equipped with an energy-dispersion X-ray spectroscopy modulus (Hitachi High Technologies Company, Tokyo, Japan) was employed to investigate the FEYNF peptide before and after incubation.

### 4.8. Molecular Docking

The molecular docking simulations were conducted on a Dell Precision workstation T7910 equipped with 32 CPU threads. The AutoDock VINA technique, available in the YASARA-Structure software package (version 20.8.23), was utilized for modeling and visualization [41,42,43,44]. Within the YASARA-Structure software, the spatial conformers in 3D of the FEYNF pentapeptide and Drew–Dickerson dodecamer were constructed using the “BuildMol” function. Specifically, the FEYNF 3D conformation incorporated the amino acids F-phenylalanyl, E-glutamyl, Y-tyrosyl, and N-asparaginyl. The dodecamer was formed using the nucleotide sequences of a sense strand 5′-CGCGAATTCGCG-3′ and a complementary anti-sense strand 3′-GCGCTTAAGCGC-5′. The 3D structures were subjected to energy minimization (for geometry optimization) at the level of molecular mechanics theory using the YASARA force field.

## Figures and Tables

**Table 1:** Values of the torsion angles (*Φ*—*Phi* and *Ψ*—*Psi*) for the two FEYNF peptide chains in the optimized dimer conformation.

FEYNF Peptide Chain	Torsion Angles ^1^					
	*Φ*_1_	*Ψ*_1_	*Φ*_2_	*Ψ*_2_	*Φ*_3_	*Ψ*_3_
Chain-1	−137.069°	144.980°	−137.509°	123.895°	−126.403°	151.016°
Chain-2	−81.123°	132.445°	−139.163°	116.446°	−43.919°	−52.269°

^1^ The determined torsion angles (dihedrals) correspond to the ones represented in Figure 1a.

## Data Availability

Data are contained within the article and supplementary material.

## Supplementary Materials

The following supporting information can be downloaded at https://www.mdpi.com/article/10.3390/gels10020086/s1. Figure S1: The gradient protocol used in HPLC analysis; Figure S2: HPLC chromatogram of the FEYNF-NH_2_ peptide following purification through solid-phase extraction, revealing a calculated purity of 99.54%. The chromatogram was generated using an injection volume of 5 µL, and the data were recorded at a detection wavelength of 215 nm. The observed peak patterns and their intensities affirm the successful purification, highlighting the peptide’s exceptional purity achieved through the solid-phase extraction process; Figure S3: Visualization of the crystals formed by the HCCA and DHB matrices (a,b) and the co-crystals with the FEYNF samples on the target (c,d). Images obtained by means of the camera equipped with the MALDI-ToF mass spectrometer. Retention times are reported based on HPLC analysis; Figure S4: FTIR spectrum of FEYNF-NH_2_ peptide evidencing the amide I–amide II spectral range and the baseline applied for data processing; Table S1: The content of secondary structures (%) of the FEYNF-NH_2_ revealed from FTIR spectrum analysis; Figure S5: TEM images of FEYNF-NH_2_ before (a) and after 20 h of incubation at physiological pH of 7.4 and a temperature of 37 °C; EDX spectra for FEYNF peptide sample before (c) and after incubation (d); Figure S6: Microscopic images of FEYNF-NH_2_ sample after Congo red staining captured under two polarization angles: 0 degrees (a), and 90 degrees (b); Table S2: Representative clusters of the docking simulation for the FEYNF@FEYNF dimer complex, highlighting the amino acid residues involved in the peptide–peptide interaction, binding energy (*E_b_*) and dissociation constant (*K_d_*) values; Table S3: Intermolecular and intramolecular interactions occurring in the FEYNF@FEYNF dimer complex, with corresponding amino acid residues involved; Figure S7: Graphical representation of (*Φj*,*Ψj*) plot (Chain 1: (*Φ*_1_,*Ψ*_1_)—red, (*Φ*_2_,*Ψ*_2_)—green, (*Φ*_3_,*Ψ*_3_)—blue; Chain 2: (*Φ*_4_,*Ψ*_4_)—cyan, (*Φ*_5_,*Ψ*_5_)—magenta, (*Φ*_6_,*Ψ*_6_)—yellow) based on determined torsion angles; Figure S8: Graphical representation of Drew–Dickerson dodecamer sequence (d(CGCGAATTCGCG)2) with molecular structures of corresponding nitrogenous bases (C-cytosine, G-guanine, A-adenine, T-tymine) and sugar-phosphate backbone (KingDraw V3.0.2.); Table S4: Representative clusters of the docking simulation for the investigated polyplex (binding energy (*E_b_*), dissociation constant (*K_d_*) and contacting residues); Figure S9: Molecular docking outcomes for the polyplex formation: (a) data for all 100 docked poses of polyplex conformation (binding energy vs. dissociation constant); (b) data for the 12 representative clusters (obtained from clustering analysis).
