# Supplementary material for: Self-Assembly of a Novel Pentapeptide into Hydrogelated Dendritic Architecture: Synthesis, Properties, Molecular Docking and Prospective Applications
Source: Gels. 2024 Jan 23;10(2):86. doi: 10.3390/gels10020086 (PMC10887771; doi:10.3390/gels10020086)
Supplement: Supplementary file 1 [file gels-10-00086-s001.zip › gels-2812516-supplementary.pdf]

gels

Supplementary materials

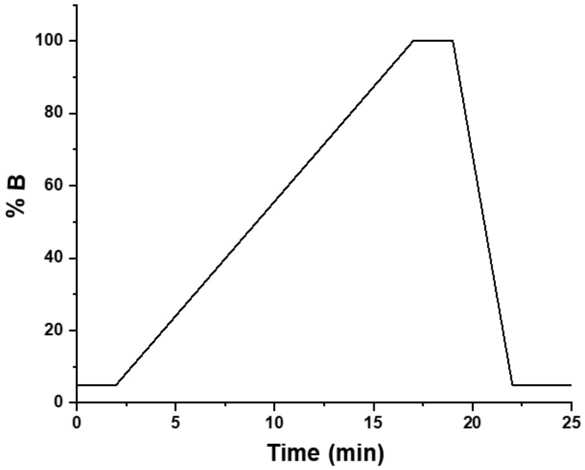

Time (min)	%B	Flow (mL/min)
0	5	1
2	5	1
17	100	1
19	100	1
22	5	1
25	5	1

Figure S1. The gradient protocol used in HPLC analysis.

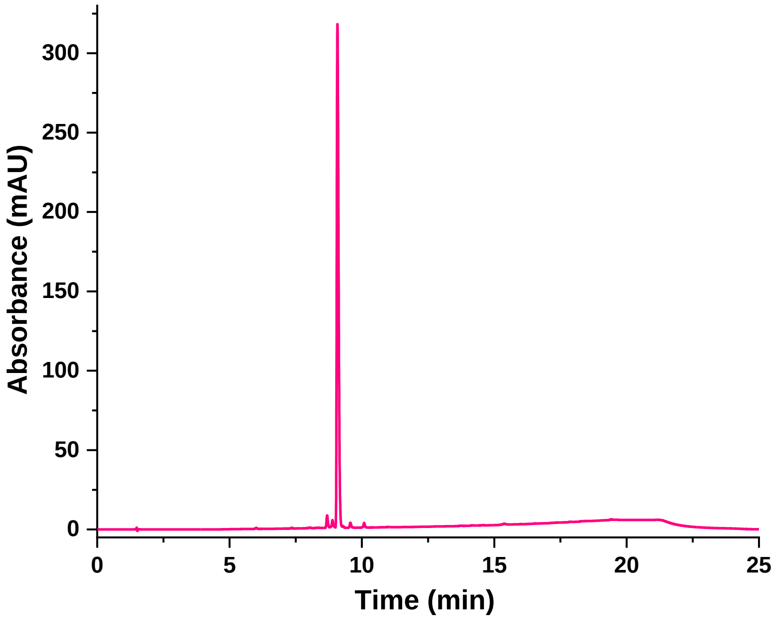

Figure S2. HPLC chromatogram of the FEYNF-NH2 peptide following purification through solid-phase extraction, revealing a calculated purity of 99.54%. The chromatogram was generated using an injection volume of 5 μL, and the data were recorded at a detection wavelength of 215 nm. The observed peak patterns and their intensities affirm the successful purification, highlighting the peptide's exceptional purity achieved through the solid-phase extraction process.

gels

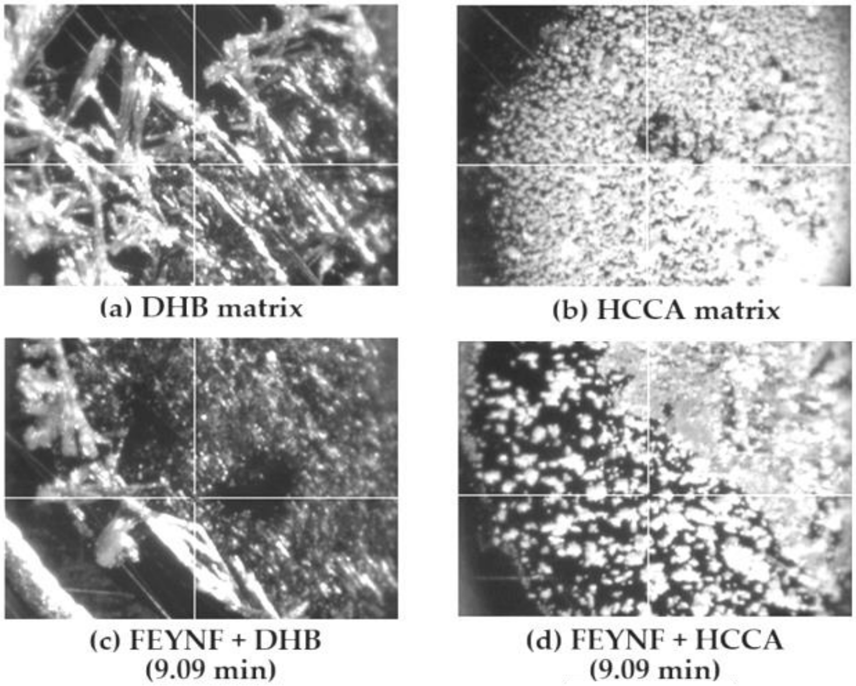

Figure S3. Visualization of the crystals formed by the HCCA and DHB matrices (a,b) and the co-crystals with the FEYNF samples on the target (c,d). Images obtained by means of the camera equipped with the MALDI-ToF mass spectrometer. Retention times are reported based on HPLC analysis.

gels

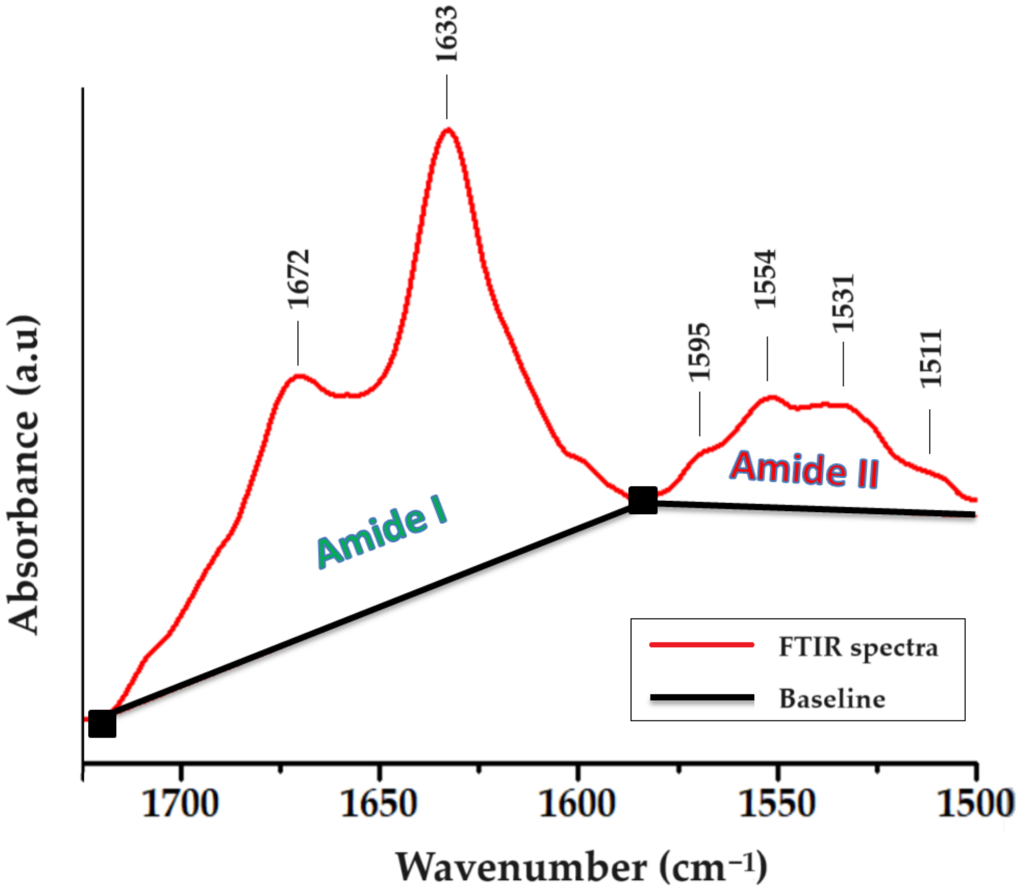

Figure S4. FTIR spectrum of FEYNF-NH_2 peptide evidencing the amide I–amide II spectral range and the baseline applied for data processing.

Table S1. The content of secondary structures (%) of the FEYNF-NH_2 revealed from FTIR spectrum analysis.

Peak no.	Structure	Wavenumber (cm^{-1})	Relative intensity (%)*
1	Antiparallel β-sheet	1694	3.12
2	β-turns	1672	16.12
3	α-helix	1657	28.01
4	β-sheet	1633	34.53
5	β-sheet aggregate strands	1613	10.56
6	Side chain	1596	7.65

* The relative intensity indicates the proportion of a component band in the total integrated area of the Amide I region.

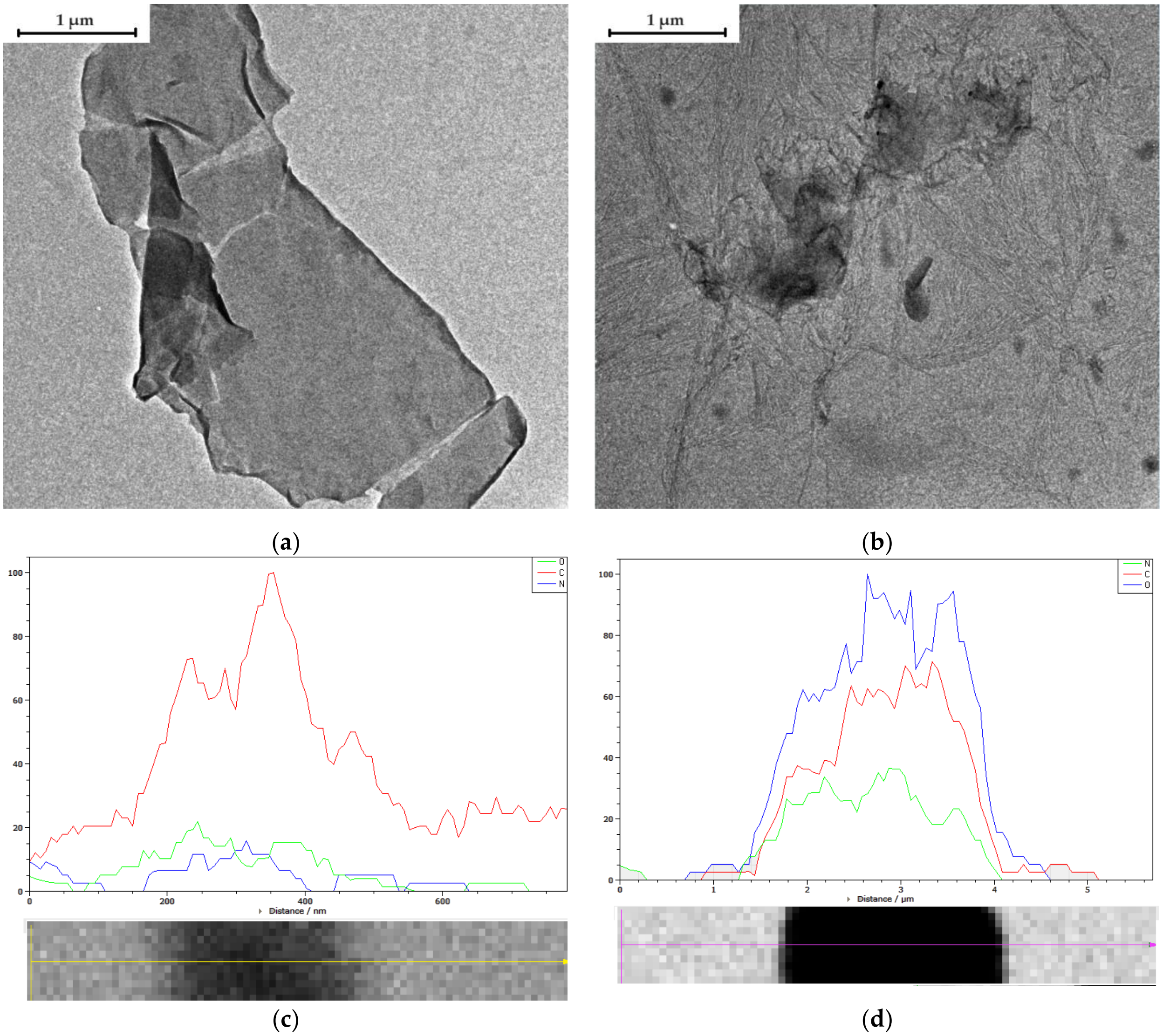

Figure S5. TEM images of FEYNF-NH_2 before (a) and after 20 h of incubation at physiological pH of 7.4 and a temperature of 37 °C; EDX spectra for FEYNF peptide sample before (c) and after incubation (d);

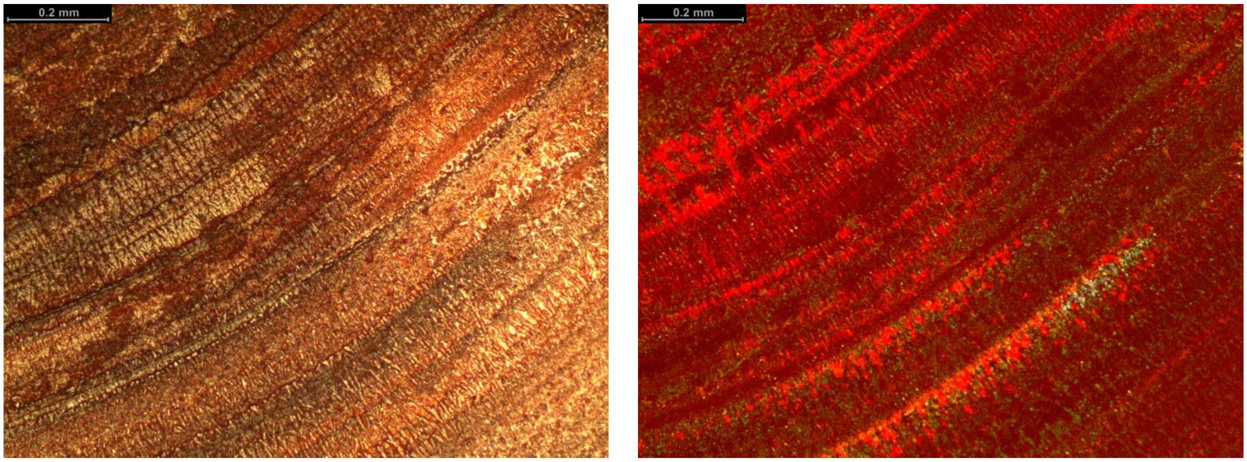

(**a**) (**b**)

Figure S6. Microscopic images of FEYNF-NH_2 sample after Congo red staining captured under two polarization angles: 0 degrees (a), and 90 degrees (b).

Table S2. Representative clusters of the docking simulation for the FEYNF@FEYNF dimer complex, highlighting the amino acid residues involved in the peptide–peptide interaction, binding energy (E_b) and dissociation constant (K_d) values

Cluster	Binding energy, (E_b, kcal/mol)	Dissociation constant (K_d, µm)	Contacting amino acid residue
1	−5.38	113.87	PHE1 GLU2 TYR3 ASN4 PHE5
2	−5.21	150.69	PHE1 GLU2 TYR3 ASN4 PHE5
3	−5.02	208.01	PHE1 GLU2 TYR3 ASN4 PHE5
4	−5.00	216.98	PHE1 GLU2 TYR3 ASN4 PHE5
5	−4.94	238.89	PHE1 GLU2 TYR3 ASN4 PHE5
6	−4.94	239.70	PHE1 GLU2 TYR3 ASN4 PHE5
7	−4.94	240.92	PHE1 GLU2 TYR3 ASN4 PHE5
8	−4.91	250.45	PHE1 GLU2 TYR3 ASN4 PHE5
9	−4.90	257.31	PHE1 GLU2 TYR3 ASN4
10	−4.87	271.59	PHE1 GLU2 TYR3 ASN4 PHE5
11	−4.81	300.53	PHE1 GLU2 TYR3 ASN4 PHE5

Table S3. Intermolecular and intramolecular interactions occurring in the FEYNF@FEYNF dimer complex, with corresponding amino acid residues involved.

Interaction type	Ligand amino acid residue	Receptor amino acid residue
H-bonds	PHE1 (O)	GLU2 (N-H)

gels

	N4 (O)(<br>-	GLU2 (O-H)<br>TYR3 (O)…ASN4 (N-H)<br>(intramolecular)
Hydrophobic interactions	PHE1	PHE1
	PHE1	TYR3
	GLU2	GLU2
	TYR3	TYR3
	TYR3	PHE5
	PHE5	ASN4
	PHE5	PHE5
π-π stacking	PHE1	PHE1
	TYR3	TYR3
	TYR3	PHE5
	PHE5	PHE5

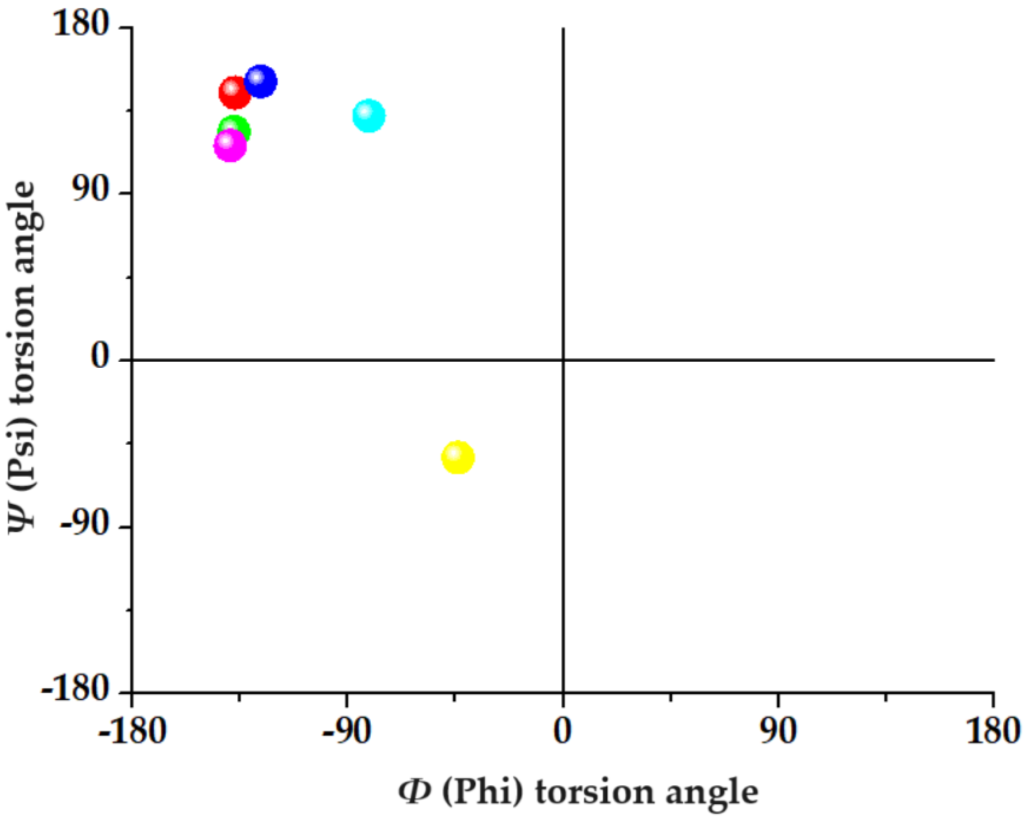

Figure S7. Graphical representation of (Φ_j,Ψ_j) plot (Chain 1: (Φ_1,Ψ_1) – red, (Φ_2,Ψ_2) – green, (Φ_3,Ψ_3) – blue; Chain 2: (Φ_4,Ψ_4) –cyan, (Φ_5,Ψ_5) – magenta, (Φ_6,Ψ_6) – yellow) based on determined torsion angles.

gels

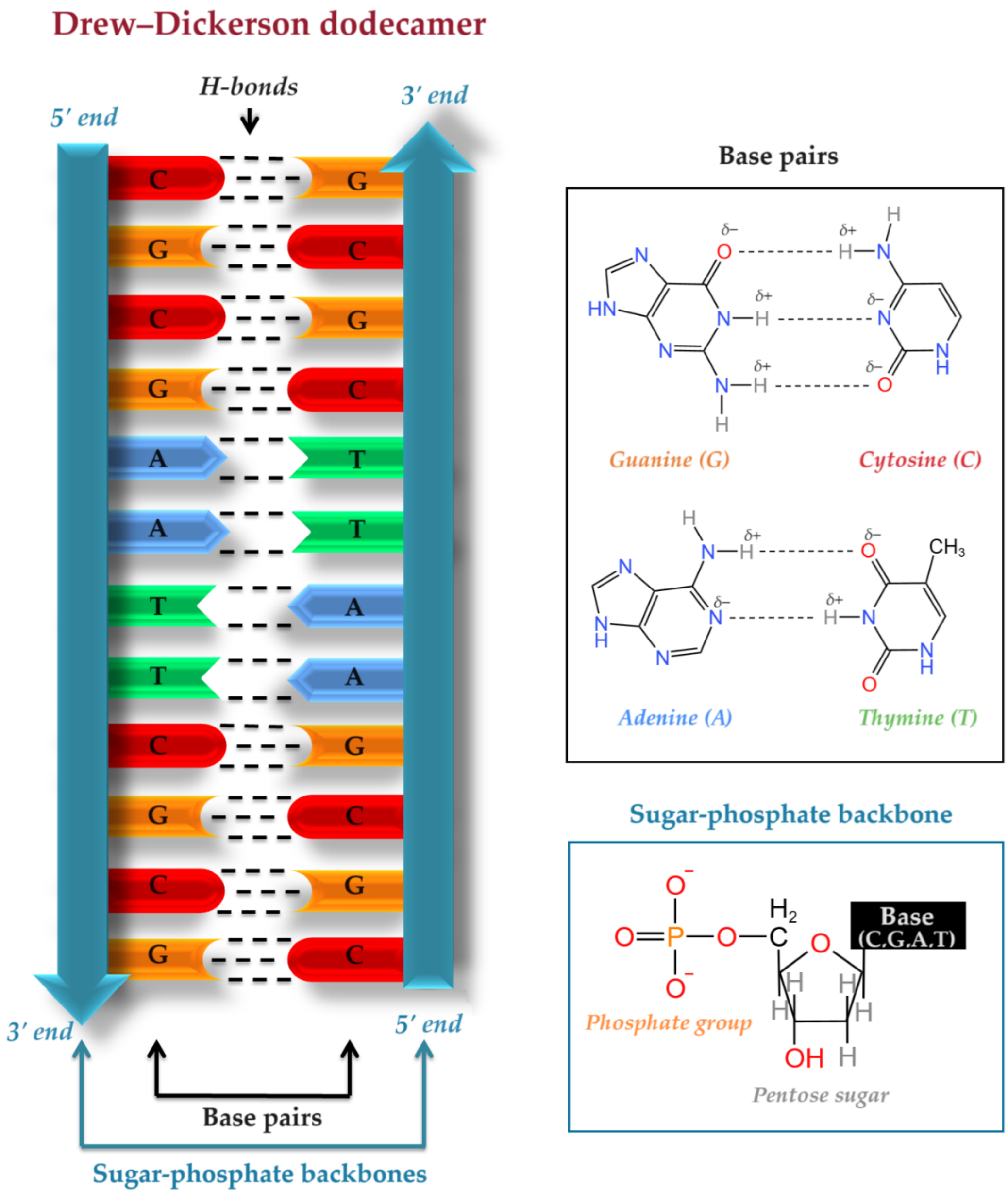

Figure S8. Graphical representation of Drew–Dickerson dodecamer sequence (d(CGCGAATTCGCG)2) with molecular structures of corresponding nitrogenous bases (C-cytosine, G-guanine, A-adenine, T-tymine) and sugar-phosphate backbone (KingDraw V3.0.2.).

gels

MDPI

Table S4. Representative clusters of the docking simulation for the investigated polyplex (binding energy (E_b), dissociation constant (K_d) and contacting residues)

Cluster	Binding energy, (E_b, kcal/mol)	Dissociation constant (K_d, nm)	Contacting receptor residue (nitrogenous base)[1]
1	−12.494	0.695	C3(a) G4(a) A5(a) A6(a) T7(a) C3(b) G4(b) A5(b) A6(b) T7(b)
2	−12.289	0.982	C3(a) G4(a) A5(a) A6(a) T7(a) C3(b) G4(b) A5(b) A6(b) T7(b)
3	−11.656	2.858	C3(a) G4(a) A5(a) A6(a) A5(b) A6(b) T7(b) T8(b) C9(b)
4	−11.241	5.758	A5(a) A6(a) T7(a) T8(a) C9(a) C3(b) G4(b) A5(b) A6(b) T7(b)
5	−10.964	9.189	C1(a) G2(a) C3(a) G4(a) A5(a) T7(b) T8(b) C9(b) G10(b) C11(b)
6	−10.908	10.100	C1(a) G2(a) C3(a) G4(a) A5(a) T7(b) T8(b) C9(b) G10(b) C11(b)
7	−10.868	10.806	T7(a) T8(a) C9(a) G10(a) C1(b) G2(b) C3(b) G4(b) A5(b)
8	−10.832	11.483	A6(a) T7(a) T8(a) C3(b) G4(b) A5(b) A6(b)
9	−10.779	12.557	C3(a) G4(a) A5(a) A6(a) T7(a) A5(b) A6(b) T7(b) T8(b)
10	−10.754	13.098	A6(a) T7(a) G2(b) C3(b) G4(b) A5(b) A6(b)
11	−10.544	18.670	G4(a) A5(a) A6(a) G4(b) A5(b) A6(b) T7(b)
12	−10.43	22.631	G4(a) A5(a) A6(a) T7(a) T8(a) C9(a) C3(b) G4(b) A5(b) A6(b)

[1] The contact residues are the nitrogenous bases citosyne (C), guanine (G), adenine (A) and Thymine (T); the base residues are found on both sugar-phosphate backbones, reffered to as (a) and (b), respectively.

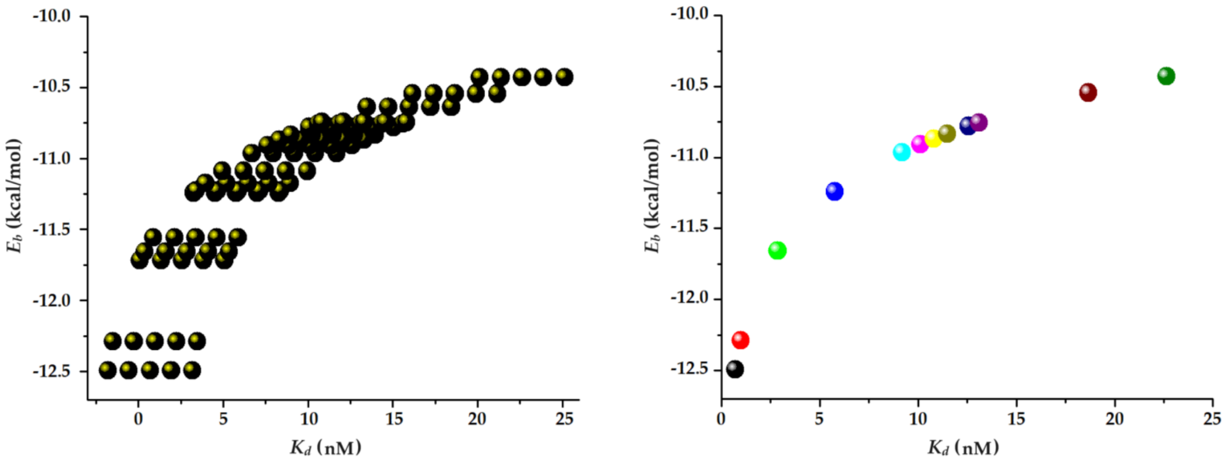

(a) (b)

Figure S9. Molecular docking outcomes for the polyplex formation: (a) data for all 100-docked poses of polyplex conformation (binding energy vs. dissociation constant); (b) data for the 12 representative clusters (obtained from clustering analysis).
